# Discovery by a proteomic approach of possible early biomarkers of drug-induced nephrotoxicity in medication-overuse headache

**DOI:** 10.1186/1129-2377-14-6

**Published:** 2013-01-30

**Authors:** Elisa Bellei, Emanuela Monari, Aurora Cuoghi, Stefania Bergamini, Simona Guerzoni, Michela Ciccarese, Tomris Ozben, Aldo Tomasi, Luigi Alberto Pini

**Affiliations:** 1 Department of Diagnostic Medicine, Clinic and Public Health, University of Modena and Reggio Emilia, Via del Pozzo 71, 41100, Modena, Italy; 2 Inter Department Headache and Drug Abuse Centre, University of Modena and Reggio Emilia, Modena, Italy; 3 Department of Biochemistry, Akdeniz University, Antalya, Turkey

**Keywords:** Medication-overuse headache, Proteomics, NSAIDs, Nephrotoxicity, SELDI-TOF-MS, Two-dimensional gel electrophoresis

## Abstract

**Background:**

Medication-overuse headache (MOH) is a chronic headache condition that results from the overuse of analgesics drugs, triptans, or other antimigraine compounds. The epidemiology of drug-induced disorders suggests that medication overuse could lead to nephrotoxicity, particularly in chronic patients. The aim of this work was to confirm and extend the results obtained from a previous study, in which we analyzed the urinary proteome of 3 MOH patients groups: non-steroidal anti-inflammatory drugs (NSAIDs), triptans and mixtures abusers, in comparison with non-abusers individuals (controls).

**Methods:**

In the present work we employed specialized proteomic techniques, namely two-dimensional gel electrophoresis (2-DE) coupled with mass spectrometry (MS), and the innovative Surface-Enhanced Laser Desorption/Ionization Time-of-Flight mass spectrometry (SELDI-TOF-MS), to discover characteristic proteomic profiles associated with MOH condition.

**Results:**

By 2-DE and MS analysis we identified 21 over-excreted proteins in MOH patients, particularly in NSAIDs abusers, and the majority of these proteins were involved in a variety of renal impairments, as resulted from a literature search. Urine protein profiles generated by SELDI-TOF-MS analysis showed different spectra among groups. Moreover, significantly higher number of total protein spots and protein peaks were detected in NSAIDs and mixtures abusers.

**Conclusions:**

These findings confirm the presence of alterations in proteins excretion in MOH patients. Analysis of urinary proteins by powerful proteomic technologies could lead to the discovery of early candidate biomarkers, that might allow to identify MOH patients prone to develop potential drug overuse-induced nephrotoxicity.

## Background

Medication-overuse headache (MOH) is a secondary cause of chronic daily headache, where headaches occur 15 or more days per month when the therapeutic agent is used excessively and on a regular basis for 3 or more months, and when headaches have developed or markedly worsened during the period of medication overuse [1]. Approximately 40% of all patients attending Headache Centers presents a chronic form of headache and 80% of these subjects excessively use symptomatic drugs which include analgesics, migraine-specific medications (such as triptans), opioids, or drugs combinations [2]. Although MOH has a prevalence of 1-2% in the general population, it represents a relevant health problem associated with significant long-term morbidity and disability [3]. MOH manifests as increased frequency and intensity of migraine attacks and as enhanced sensitivity to stimuli that elicit migraine episodes [4]. Moreover, there are evidences to support a role for genetic factors in the development of MOH [5]. It is now recognised that MOH has characteristic clinical features, a clear biological basis and causes considerable morbidity [6,7]. A recent study concerning the epidemiology of drug-induced disorders has demonstrated that medication overuse could lead to nephrotoxicity and potential renal damage [8]. Particularly, drug-associated nephrotoxicity accounts for 18-27% of all acute kidney injury cases in US and medications can affect all aspects and every part of the kidney’s structure by different mechanisms of renal dysfunctions. The objective of this work was to extend and confirm the results from a previous study [9], in which we analyzed the urinary proteome of MOH patients overusing non-steroidal anti-inflammatory drugs (NSAIDs), triptans and mixtures, in comparison with healthy non-abusers individuals as controls, with the aim to identify significantly differences in excreted proteins induced by the excessive consumption of drugs and potentially related to nephrotoxicity. In the present study, other than to increase the number of patients and controls, we adopted further specialized proteomic techniques, namely two-dimensional gel electrophoresis (2-DE) coupled with mass spectrometry (MS), and the innovative and sensitive Surface-Enhanced Laser Desorption/Ionization Time-of-Flight mass spectrometry (SELDI-TOF-MS), with the main purpose to discover new proteomic profiles associated with MOH condition.

## Methods

### Patients and controls selection

Eighty-seven MOH patients were recruited by the “Headache and Drug Abuse Centre” of the University-Hospital of Modena and Reggio Emilia, Italy. As illustrated in Table 1, they were assigned to three different groups according to the type of primary overused drug: 27 patients who abused of one or more types of NSAIDs (aged 36–62 years, mean 49.2 ± 7.8); 31 who abused exclusively triptans (aged 32–65 years, mean 46.0 ± 9.3); and 29 patients assuming two or more drugs simultaneously or mixtures, i.e. combination of indomethacin, caffeine and perchlorperazine (aged 35–66 years, mean 51.8 ± 9.9). Mixtures are the most utilized self-medication for the treatment of migraine in Italy, so that several patients suffering from chronic headache overuse this symptomatic medication in the attempt to control their daily headache attacks [10]. Each group was matched for age, gender, MOH duration, days with headache per month, and about daily drug intake. Thirty healthy volunteers (aged 42–58 years, mean 48.1 ± 5.0), were also enrolled and used as controls. Control group did not differ in age or gender in respect of MOH patient groups. For all study participants exclusion criteria included proved kidney diseases and urogenital tract dysfunctions, other important acute or chronic medical illness (such as heart failure, liver injury, malignancy, inflammatory diseases), elevated levels of serum creatinine and assumption of counter medicines (other than NSAIDs and triptans for MOH patients). Before starting the study, haematological screening and chemical-physical examination of urine were performed in all participants, obtaining values in the normal range. Informed consent was provided from each subject, following an exhaustive description of the study procedures and objectives. The study was approved by the Ethical Committee research of the University-Hospital of Modena, and it was conducted in strict conformity with the Declaration of Helsinki.

**Table 1:** Details of MOH patients and control subjects

	**NSAIDs group**	**Triptans group**	**Mixtures group**	**Control group**
No. of subjects	27	31	29	30
Age *(years)*	49.2 ± 7.8	46.0 ± 9.3	51.8 ± 9.9	48.1 ± 5.0
Gender *(F/M)*	23/4	28/3	27/2	26/4
MOH duration *(years)*	8.7 ± 6.0	7.9 ± 5.5	9.4 ± 6.6	N/A
Days with headache/month	25.3 ± 3.5	26.5 ± 4.6	24.4 ± 5.1	N/A
Daily drug intake	2.0 ± 1.3	1.4 ± 0.6	1.8 ± 0.8	N/A

### Preparation of urine samples

Second void morning urine samples (midstream) were collected and kept on ice until centrifugation (800 × g, 10 min, 4°C) to remove cell debris and other solid materials. Since human urine has a very dilute protein concentration and a high-salt content, which interferes with 2-DE analysis, the supernatants were concentrated and desalted using 3 kDa MW cut-off filter devices (Millipore). Moreover, to avoid transient increase in protein excretion, all subjects were asked to refrain from unusual physic activity the day before urine collection. The total protein concentration was estimated by the spectrophotometric Bradford’s method [11], and finally the samples were divided into aliquots and stored at −80°C until use, to prevent proteolysis and bacterial growth.

### Two-dimensional gel electrophoresis (2-DE)

The concentrated/desalted urine sample (100 μg of total protein) was subjected to isoelectrofocalization (IEF), as previously described [12]. Briefly, after IEF, the second-dimension separation was performed using 8-16% acrylamide gradient gels and the urinary proteins were visualized according to a silver nitrate staining protocol. Gel images were acquired by a calibrated densitometer (Bio-Rad GS800) and analyzed by the powerful image analysis software program PDQuest (version 7.3.1, Bio-Rad) to identify differently expressed proteins in the different groups. The PDQuest software compares images of 2-DE gels to determine differential protein expression and accurately identifies increased or decreased proteins on the basis of spots staining intensity. When spots were detected, the original gel image was normalized by a filtration procedure and the 3-D Gaussian spots were created. Subsequently, a standard gel image (as a master map), made by merging the Gaussian images of each NSAIDs patient, was matched with the primary gels images obtained from healthy subjects, triptans and mixtures, generating a match set. The protein spots in a comparison set of gels were quantitatively, qualitatively, and statistically analyzed, selecting for the identification by MS the protein spots with an expression difference higher than 1.5-fold.

### Proteins identification by mass spectrometry

Significant spots were selected, excised manually from the gels and “in-gel” digested with trypsin, to obtain peptide fragments ready to be examined by MS, as already reported in detail [13]. Briefly, protein spots were first de-stained and then reduced with dithiothreitol DTT and alkylatated with iodoacetamide. Subsequently, dried samples were digested with trypsin by incubation overnight at 37°C. After digestion, the peptides were first extracted and then concentrated in a vacuum drier. The peptide mixtures were analyzed using the 6520 Accurate-Mass Quadrupole-Time of Flight Liquid Chromatography-Mass Spectrometry (Q-TOF LC/MS, Agilent Technologies, CA, USA), as previously fully described [14].

### SELDI-TOF-MS analysis

Urine samples were analyzed on CM10 weak-cation exchange ProteinChip arrays, according to the manufacturer’s instructions. In brief, urine samples were mixed with the indicated array-specific binding buffer and loaded randomly in duplicate onto the pre-equilibrated ProteinChip surface, in order to minimize any error source. The matrix solution (saturated sinapinic acid in 0.5% trifluoroacetic acid/50% acetonitrile) was applied to each spot twice and then the ProteinChip arrays were analyzed by the ProteinChip reader Series 4000 (Bio-Rad), applying the reading protocols optimized for low and high mass to charge (m/z) range. Furthermore, to improve reproducibility, all steps were automated using the robotic instrument for liquid handling Biomek 3000 Laboratory Automation Workstation, (Beckman Coulter).

### Statistics

All data are provided as mean ± standard deviation (SD). Statistical significance was tested using the Student’s *t*-test (two-tail), considering a p-value less than 0.05 as statistically significant. Regarding SELDI-TOF analysis, statistics was performed by the ProteinChip Data Manager 3.0 software (Bio-Rad). The spectra were baseline subtracted, normalized by total ion current in the range of interest and finally mass aligned. Supervised clustering was carried out using the following settings: 5 times signal-to-noise (S/N) ratio and 20% min peak threshold in the first pass for peaks identification; 2 times S/N ratio on the second pass for cluster completion. Spectra analysis was performed using the unpaired Student’s *t*-test (p-value <0.05). Moreover, to test the overall quality of the assay, a pooled sample of urine was used as quality control (QC), to assess the reproducibility during all experiment. QC sample replicates were used to calculate the median coefficient of variation (CV) for each detected peak.

## Results

### Urine analysis by 2-DE and protein MS identification

**Figure 1:**
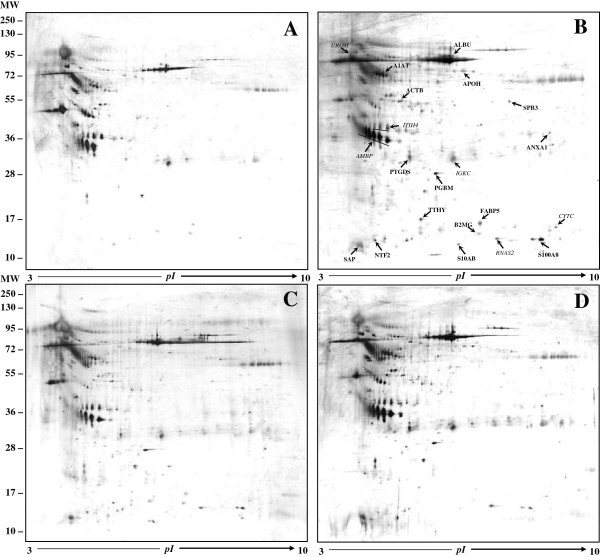
**Comparison among 2-D protein profiles obtained from each group.** (**A**) Control subjects, (**B**) NSAIDs, (**C**) triptans and (**D**) mixtures abusers. In gel image from NSAIDs patients (**B**), the differentially expressed protein spots are indicated with arrows (in Italics: proteins previously identified; in Bold: new detected proteins). The protein entry names correspond to those listed in Table 2.

**Figure 2:**
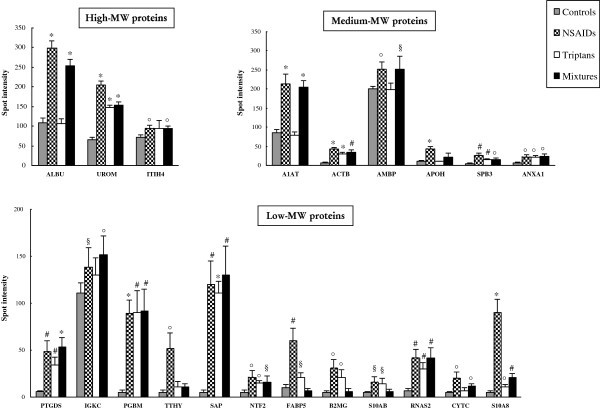
**Histograms showing the proteins differentially expressed in MOH patients compared with control subjects, at high-, medium- and low-MW.** Each bar represents the normalized and averaged value of spot volume intensity ± standard deviations (SD), obtained by the PDQuest image analysis software. (*p<0.0001; #p<0.001; °p<0.01; §p<0.05 *vs* controls).

In Figure 1 are illustrated the representative 2-D gels obtained from controls (Figure 1A) and MOH patients (Figures 1B-D). Analyzing the differently abundant protein spots by spot intensity quantification using the PDQuest software, we found a total of 21 over-expressed proteins in patients than in control subjects (considering an expression difference >1.5-fold). These proteins, identified by Q-TOF LC/MS analysis and listed in Table 2, are also indicated with arrows and their entry names in NSAIDs gel image (Figure 1B, bold). In Table 2 the first column reports the protein entry names derived from the UniProt knowledge database and the second column provides the full common name of the protein. Column 3 refers to the UniProt database primary protein accession number and column 4 indicates the specific gene name. Columns 5 and 6 denote the theoretical MW and the proteins isoelectric point, respectively. In column 7 are shown the ion scores expressed as the probability that the observed match between the experimental data and the database sequence is a random event. Single ions scores >25 indicate identity or extensive homology. Column 8 reports the query values, namely the total number of peptides that match the identified protein, and the subsequent column indicates the sequence coverage, that is the percentage of amino acids sequenced for each detected peptide. The final 3 columns summarize the significant protein over-expression in each MOH patients group compared with controls. Interestingly, 6 proteins (UROM, ITIH4, AMBP, IGKC, RNAS2, CYTC) that were identified as differentially expressed in our previous study [9], were confirmed in this work by 2-DE analysis. In addition, as evident in Figure 2 and outlined in Table 2, several novel significant changes in protein expression were revealed in each range of MW. Specifically, serum albumin (ALBU) at high-MW and alpha-1-antitrypsin (A1AT) at medium-MW, were found significantly over-excreted only in NSAIDs and mixtures abusers, while other three medium-MW proteins, namely actin, cytoplasmic1 (ACTB), serpin B3 (SPB3) and annexin A1 (ANXA1) resulted over-expressed in all MOH groups compared with controls. Finally, apolipoprotein H (APOH) was found significantly increased only in NSAIDs. The greatest differences were found at low-MW. In fact, 5 proteins were significantly over-expressed in all MOH groups: prostaglandin-H2-D-isomerase (PTGDS), perlecan fragment (PGBM), proactivator polypeptide (SAP), nuclear transport factor 2 (NTF2), and protein S100-A8 (S10A8). Three proteins resulted over-expressed only in NSAIDs and triptans: fatty acid-binding protein (FABP5), beta-2-microglobulin (B2MG), and protein S100-A11 (S10AB), while transthyretin (TTHY) was increased only in NSAIDs patients. Furthermore, many differential protein spots distributed throughout the gel map were identified as ALBU fragments (data not shown). It is interesting to note that NSAIDs abusers showed a significant over-expression for all the 15 detected proteins, while triptans and mixtures patients presented a total of 11 and 10 increased proteins, respectively. At last, we performed an automatic spots count for each group, using the PDQuest software after appropriate parameters calibration. As summarized in Table 3, we obtained a significantly elevated number of total protein spots only in NSAIDs and mixtures groups, but not in triptans group, with an increase *vs* controls of 50%, 41% and 13%, respectively.

**Table 2:** Differentially expressed proteins identified by Q-TOF LC/MS

**Protein Entry name**^**a**^	**Protein full name**	**Acc. no.**^**b**^	**Gene name**	**Theor. MW**^**c**^***(kDa)***	**p*I***	**Score**^**d**^	**Pept.**^**e**^	**Seq. cov.*(%)***^**f**^	**Over-expression*vs*controls**^**g**^		
									**NSAIDs Triptans Mixtures**		
*High-MW proteins*											
**ALBU**	Serum albumin	P02768	ALB	66.5	5.67	156	12	53	x	NS	x
UROM^(*)^	Uromodulin	P07911	UMOD	69.7	4.96	78	23	45	x	x	x
ITIH4^(*)^	Inter-α-trypsin inhibitor heavy chain H4	Q14624	ITIH4	70.6	5.92	37	5	14	x	NS	x
*Medium-MW proteins*											
**A1AT**	Alpha-1-antitrypsin	P01009	AAT	46.9	5.37	300	37	20	x	NS	x
**ACTB**	Actin, cytoplasmic1	P60709	ACTB	42.1	5.29	49	20	7	x	x	x
AMBP^(*)^	Alpha-1-microglobulin	P02760	AMBP	39.9	6.13	261	41	29	x	NS	x
**APOH**	Apolipoprotein H	P02749	APOH	38.3	8.37	48	12	26	x	NS	NS
**SPB3**	Serpin B3	P29508	SCCA	44.6	6.35	20	4	16	x	x	x
**ANXA1**	Annexin A1	P04083	ANX1	38.6	6.64	10	8	8	x	x	x
*Low-MW proteins*											
**PTGDS**	Prostaglandin-H2-D-isomerase	P41222	PTGDS	18.7	8.37	152	25	30	x	x	x
IGKC^(*)^	Ig kappa chain C region	P01834	IGKC	11.8	5.58	130	14	50	x	NS	x
**PGBM**	Perlecan (fragment)	P98160	HSPG2	479.2	6.06	405	25	7	x	x	x
**TTHY**	Transthyretin	P02766	TTR	15.9	5.52	32	19	32	x	NS	NS
**SAP**	Proactivator polypeptide	P07602	PSAP	9.11	4.22	87	6	6	x	x	x
**NTF2**	Nuclear transport factor 2	P61970	NUTF2	14.6	5.10	50	8	11	x	x	x
**FABP5**	Fatty acid-binding protein	Q01469	FABP5	15.5	6.82	83	18	40	x	x	NS
**B2MG**	Beta-2-microglobulin	P61769	B2M	11.7	6.07	27	4	20	x	x	NS
**S10AB**	Protein S100-A11	P31949	S100A11	11.8	6.56	96	16	23	x	x	NS
RNAS2^(*)^	Non-secretory ribonuclease	P10153	RNASE2	18.9	9.10	67	8	19	x	NS	x
CYTC^(*)^	Cystatin-C	P01034	CST3	13.3	8.75	60	13	26	x	NS	x
**S10A8**	Protein S100-A8	P05109	S100A8	10.8	6.51	572	170	68	x	x	x

^a^Protein entry name from UniProt knowledge database. ^(*)^Proteins previously identified as differentially expressed between MOH patients and controls in our previous study.

^b^Primary accession number from UniProt database.

^c^Theoretical protein molecular weight.

^d^The highest scores obtained with MASCOT search engine.

^e^Total number of peptides that matching the identified proteins.

^f^Sequence coverage: percentage of amino acids sequenced for each detected protein.

^g^Significant (x) and not significant (NS) over-expression in each MOH group respect to controls.

**Table 3:** Quantitative analysis of total protein spots detected by 2-DE and total protein peaks detected by SELDI-TOF analysis

**Groups**	**N**^**o**^**. of spots (2-DE)**	**Increase*vs*controls (%)**	**N**^**o**^**. of peaks (SELDI-TOF)**	**Increase*vs*controls (%)**
Controls	357 ± 22	N/A	34 ± 2.9	N/A
NSAIDs	536 ± 14^**^	50	53 ± 3.3^*^	56
Triptans	403 ± 29	13	40 ± 4.1	18
Mixtures	502 ± 18^**^	41	50 ± 2.6^*^	47

Spots and peaks numbers are expressed as means ± SD. Statistical significance was tested using unpaired Student’s *t*-test (**p<0.001; *p<0.01 *vs* controls).

### SELDI-TOF-MS protein profiles

**Figure 3:**
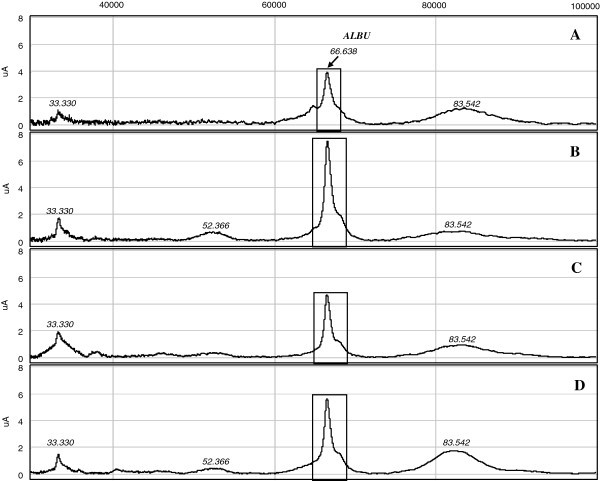
**High m/z SELDI-TOF spectra generated from urine analysis.** In each image is evident the prevalence of the ALBU peak (enclosed in rectangles). (**A**) Control subjects, (**B**) NSAIDs, (**C**) triptans and (**D**) mixtures abusers.

**Figure 4:**
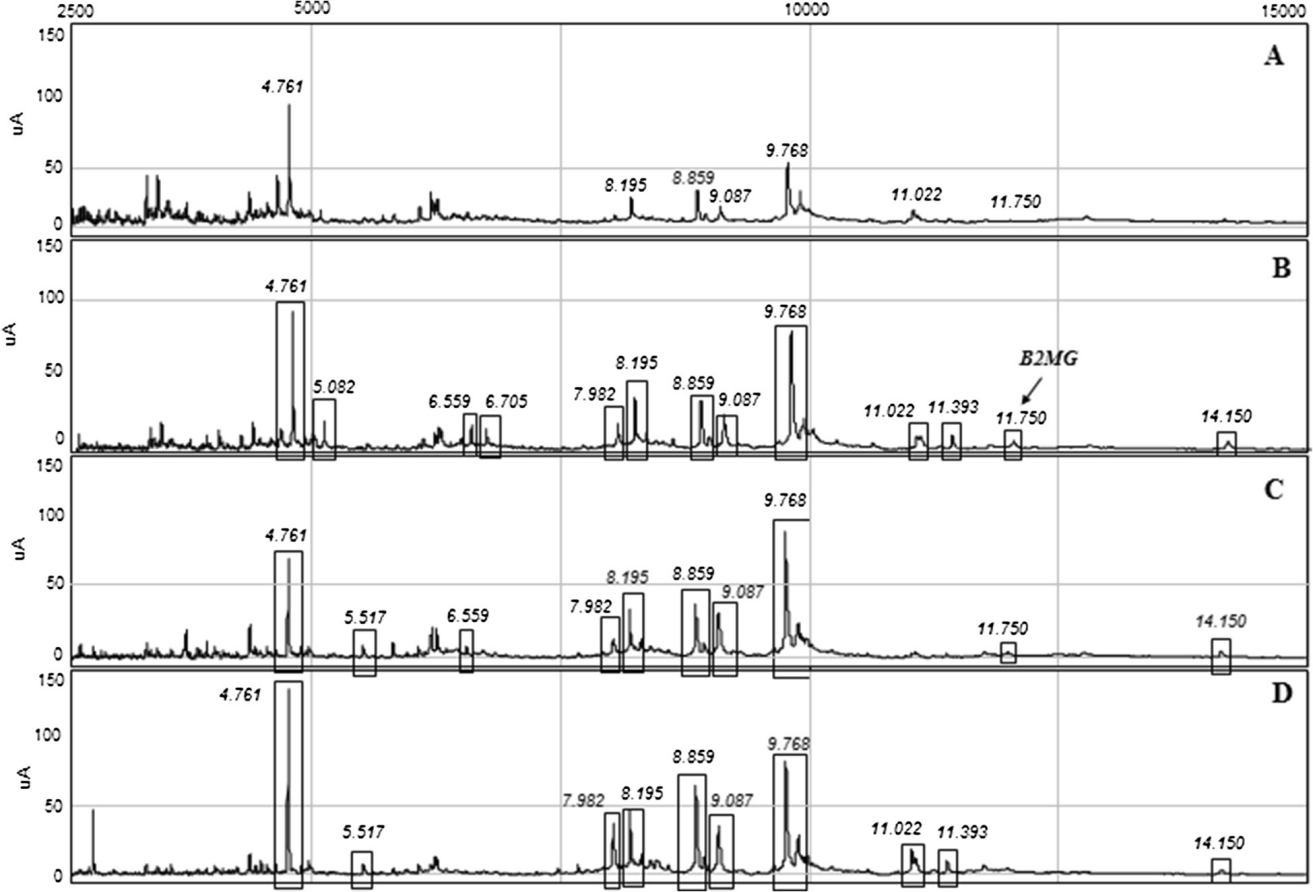
**Low m/z SELDI-TOF spectra.** In rectangles are enclosed the differential protein peaks detected in MOH patients, namely (**B**) NSAIDs, (**C**) triptans and (**D**) mixtures abusers, respect to control subjects (**A**). With arrow is indicated the B2MG peak, at m/z value of 11.750.

Urine protein profiles were generated by SELDI-TOF-MS analysis, both in the high (Figure 3) and low m/z range (Figure 4). In particular, at high m/z all spectra showed the ALBU peak (66.638 m/z) as predominant, since this is one of the most abundant protein in urine. Because of its abundance, ALBU saturated the ProteinChip array surface, compromising the detection of the other proteins present in the same m/z interval. This is the reason for the poor presence of protein peaks in these spectra (Figure 3). The relative intensity of the ALBU peak was higher in NSAIDs (Figure 3B) and mixtures patients (Figure 3D), with a measured mean intensity of 15.86 and 14.04, respectively, compared with triptans (9.10) (Figure 3C) and controls (10.77) (Figure 3A). In Figure 4 are illustrated the urinary spectra ranging from 2.500 to 15.000 m/z. Their examination highlighted different cluster of protein peaks (enclosed in rectangles) between patients and controls, with higher intensities in MOH patients groups. To cite an example, the B2MG peak (m/z 11.750) showed a relative intensity of 2.21 in controls (Figure 4A), more than double in triptans (5.45) (Figure 4C) and 3 times greater in NSAIDs (6.71) (Figure 4B). We also calculated the total number of protein peaks detected in each group. As evidenced in Table 3, only NSAIDs and mixtures patients showed a significantly higher number of protein peaks compared to controls, with a percentage increase of 56% and 47%, respectively. These results were strictly in accordance with those obtained by the 2-DE spots count.

## Discussion

This work extends and completes a previous proteomic study concerning the overuse of antimigraine drugs in MOH patients and the possible risk to develop nephrotoxicity [9]. Urinary proteomics is among the most rapidly growing subdisciplines of proteomics applied to biomedical research and represent one of the key emerging technologies to discover new biomarkers for renal disease [15,16]. In the present study we applied powerful proteomic methods for protein profiling and biomarker discovery, i.e. 2-DE together with MS and SELDI-TOF-MS. Urine analysis by 2-DE confirmed 6 proteins previously identified as differentially expressed and, in addition, revealed 15 new differential proteins in MOH patients respect to control subjects (Figure 1B, bold), in each range of MW (Figure 2 and Table 2). In particular, at high-MW we observed the over-expression of ALBU in NSAIDs and mixtures patients. This finding, even if it could be reasonably expected, has never been shown in migraineurs patients, which suffer from a neurological disorder in the absence of any tissue injury; particularly, these patients do not manifest any other disease or physical dysfunction that could be related to the observed changes in protein expression, but purely they abuse drugs from several years. From this point of view, the medication overuse can be considered the pivotal cause of the altered protein excretion. Traditionally, the occurrence of albuminuria has been held as a marker of altered glomerular permeability, and the use of ALBU excretion has been well established as a diagnostic and prognostic marker to evaluate the severity degree of glomerular damage in the progression of chronic kidney disease [17]. Moreover, in urine of MOH abusers we found also several ALBU fragments. This could be explained because during glomerular filtration (particularly in the course of acute renal injury) ALBU undergoes fragmentation through the activation of intrarenal proteases, producing modified forms and smaller peptides (<10 kDa) that are excreted in urine [18]. Moreover, in support of this hypothesis, we found also increased levels of some proteases, such as A1AT, which proteolytic activity has been observed in tubulo-interstitial damage [19], other than SAP and SPB3, that may act as hydrolases. Therefore, we might suppose the occurrence of a “proteolytic attack” against ALBU in urine of MOH patients, causing its extensive degradation. At medium-MW we found 2 proteins significantly increased in all MOH abusers, namely ACTB and ANXA1, and the over-expression of APOH only in NSAIDs group. ACTB is a component of the cytoskeleton and is a mediator of the internal cell motility, while ANXA1 is implicated in several processes, including apoptosis and specialized renal functions [20]. About APOH, despite its relatively high-MW, it is excreted in elevated quantity by patients with primary renal tubular disorders [21]. In general, high-MW proteins (such as ALBU) are used as markers of glomerular filtration and low-MW proteins (traditionally B2MG) are markers of tubular damage; both glomerular and tubular damages are followed by renal dysfunctions [22]. The increase in urinary excretion of APOH and low-MW proteins in subjects with plasma creatinine concentrations within the reference range and normal urinalysis, as MOH patients enrolled in our study, may be explained by an overflow mechanism. One possibility is that there may be competition from ALBU and other proteins for reabsorption by the proximal tubule cells. An alternative probable explanation is that it results from diminution in the ability of the proximal tubule cells to bind and incorporate these proteins into endocytic vesicles or to hydrolyze them within lysosome. In our study we detected significantly increased excretion of B2MG in both NSAIDs and triptans groups. This protein (as well as the majority of the low-MW ones), is readily and freely filtered through the glomerulus and normally almost completely reabsorbed and destroyed by proximal tubular cells, so that only 0.3% of the filtered protein is found daily in the urine. Impairment of B2MG tubular uptake results in a raised urinary excretion, so B2MG level in urine has been taken as marker of proximal tubular dysfunctions [23]. We revealed increased excretion of ALBU and B2MG in MOH abusers either by 2-DE analysis that with SELDI-TOF-MS (Figure 3 and Figure 4, respectively). This latter is a versatile high-throughput technique able to provide a rapid protein expression profile from a variety of complex biological materials [24]. Analyzing MOH patients and controls urine samples by SELDI-TOF, considerable differences in protein profiles emerged within groups, both in terms of quality (Figure 3 and Figure 4), and quantity (Table 3). In fact, as illustrated in Table 3, the total number of protein peaks detected was significantly higher in NSAIDs and mixtures patients. These results are in accordance with those obtained by the 2-DE spots count, showing a strict overlap of percentage increase values. Interestingly, total urinary proteins (together with B2MG and CYTC, also identified in our studies), have been described as nephrotoxicity markers to detect drug-induced kidney damage [25]. Another low-MW protein over-excreted in all MOH patients groups was the PTGDS, also known as prostaglandin-D_2_ synthase or β-trace protein. Prostaglandins play a major role in renal functions and development. Elevated levels of serum PTGDS, then physiologically eliminated via the kidneys, have been found in patients with impaired renal function [26], while urinary PTGDS excretion was found significantly increased in patients who were given long-term administration of gentamicin [27]. Other low-MW proteins over-expressed were: PGBM (fragment), a proteoglycan present in the glomerular basement membrane that provides a charge-selective barrier for glomerular filtration [28]; NTF2, a transport protein; S10A8, a calcium-binding regulatory protein (like S10AB), that plays a role as pro-inflammatory mediator in acute and chronic inflammation, other than to be differentially expressed in various tumor types. The hormone-binding protein TTHY (increased only in NSAIDs abusers), is mainly synthesized in the liver and its defects are the causes of amyloidosis. Insights into disease revealed that amyloid renal deposits commonly occur, together with kidney damage and proteinuria [29]. Finally, we found an over-expression of FABP5. This is an intracellular fatty acid carrier protein predominantly expressed in human proximal tubules. Several clinical studies demonstrated that in kidney disease the expression of FABP was increased and urinary excretion was accelerated, reflecting the disease progression [30]. For these reasons FABP has been proposed as an excellent biomarker for predicting and monitoring deterioration of renal function and for early detection of kidney injury [31]. The mechanisms of nephrotoxicity can vary between agents, including indirect or direct effects, that may be localized to a particular anatomical site. For this reason, there is much interest in the discovery of new biomarkers, that might provide a sensitive and rapid tool for the diagnosis of possible acute kidney injury after drug exposure, and thus allow earlier intervention with remedial therapy. In recent years, a broad range of serum and urinary enzymes and proteins have been proposed as possible early biomarkers of drug-induced nephrotoxicity [32], also through the use of proteomic methods [33]. In the present study, utilizing a proteomic approach, we evidenced an over-expression of a definite pattern of urinary proteins largely associated with various renal dysfunctions, particularly in NSAIDs abusers; this finding supports our previous results. Moreover, NSAIDs and mixtures abusers showed a significantly elevated number of total proteins, with a mean increase of around 50% respect to controls.

## Conclusions

To summarize, regarding the possibility of drug-induced nephrotoxicity, we confirmed no particular risk only associated with the use of triptans. On the other hand, it is important to emphasize that, so far, none of the MOH patients participating to this study has shown any clinical sign or symptoms of kidney impairment; furthermore, their serum creatinine levels are in the normal range. It is specifically for these reasons that the over-expressed proteins that we found in urine of these abusers might represent potential early candidate biomarkers of kidney injury. Based on the results obtained in this work, we point out that it is important to monitor the renal function of MOH patients, especially for NSAIDs and mixtures abusers, because they might be prone to gradually progress toward different renal syndromes. In this way, an earlier detection of possible acute kidney dysfunctions using a distinctive panel of protein biomarkers, each informing on the integrated aspects of glomerular, tubular and interstitial function, might provide an opportunity to minimize the potential risk to develop severe or persistent renal damages also in presence of a normal clinical pattern.

## Abbreviations

2-DE: two-dimensional gel electrophoresis;MS: mass spectrometry;SELDI-TOF-MS: Surface-Enhanced Laser Desorption/Ionization Time-of-Flight mass spectrometry;ALBU: albumin;A1AT: alpha-1-antitrypsin;ACTB: actin, cytoplasmic 1;SPB3: serpin B3;ANXA1: annexin A1;APOH: apolipoprotein H;PTGDS: prostaglandin-H2-D-isomerase;PGBM: perlecan;SAP: proactivator polypeptide;NTF2: nuclear transport factor 2;S10A8: protein S100-A8;FABP5: fatty acid-binding protein;B2MG: beta-2-microglobulin;S10AB: protein S100-A11;TTHY: transthyretin

## Competing interests

The authors declare that there is no conflict of interest.

## Authors’ contributions

EB designed the study, performed bi-dimensional gel electrophoresis and wrote the entire manuscript. EM carried out SELDI-TOF-MS analysis and performed statistical analysis. AC performed protein identification by mass spectrometry and helped in SELDI-TOF-MS experiments. SB carried out samples preparation for mass spectrometry analysis. SG and MC were responsible for patients enrollment and sample collection. TO and AT provided useful advices to improve performance, helped to draft the manuscript and finally revised it. LAP participated in the design of the study and supervised the work. All authors read and approved the final manuscript.
